# The Therapeutic Potential of the Anticancer Activity of Fucoidan: Current Advances and Hurdles

**DOI:** 10.3390/md19050265

**Published:** 2021-05-10

**Authors:** Jun-O. Jin, Pallavi Singh Chauhan, Ananta Prasad Arukha, Vishal Chavda, Anuj Dubey, Dhananjay Yadav

**Affiliations:** 1 Shanghai Public Health Clinical Center & Institutes of Biomedical Sciences, Shanghai Medical College, Fudan University, Shanghai 201508, China; 2 Department of Medical Biotechnology, Yeungnam University, Gyeongsan 38541, Korea; 3 Research Institute of Cell Culture, Yeungnam University, Gyeongsan 38541, Korea; 4 Amity Institute of Biotechnology, Amity University Madhya Pradesh, Gwalior 474005, India; pschauhan@gwa.amity.edu; 5 Comparative Diagnostic and Population Medicine, College of Veterinary Medicine, University of Florida, Gainesville, FL 32608, USA; ananta.arukhaa@gmail.com; 6 Division of Anaesthesia, Sardar Women’s Hospital, Ahmedabad 380004, Gujarat, India; chavdavishal2@gmail.com; 7 Department of Chemistry, ITM Group of Institutions, Gwalior 475005, India; anujdubey.mail@gmail.com

**Keywords:** anticancer activity, fucoidan, tumor growth, cytotoxic effects, brown algae

## Abstract

Several types of cancers share cellular and molecular behaviors. Although many chemotherapy drugs have been designed to weaken the defenses of cancer cells, these drugs may also have cytotoxic effects on healthy tissues. Fucoidan, a sulfated fucose-based polysaccharide from brown algae, has gained much attention as an antitumor drug owing to its anticancer effects against multiple cancer types. Among the anticancer mechanisms of fucoidan are cell cycle arrest, apoptosis evocation, and stimulation of cytotoxic natural killer cells and macrophages. Fucoidan also protects against toxicity associated with chemotherapeutic drugs and radiation-induced damage. The synergistic effect of fucoidan with existing anticancer drugs has prompted researchers to explore its therapeutic potential. This review compiles the mechanisms through which fucoidan slows tumor growth, kills cancer cells, and interacts with cancer chemotherapy drugs. The obstacles involved in developing fucoidan as an anticancer agent are also discussed in this review.

## 1. Introduction

Genetic engineering and biopharmaceutical research conducted on polysaccharide biomacromolecules has revealed that acidic polysaccharides from aquatic sources act as curative agents. In this respect, fucoidan, a complex polysaccharide found in certain species of brown seaweed, has shown promising results [1,2,3]. Brown macroalgae, such as gulfweed, are a class of seawater plants that are extensively dispersed in numerous cold marine regions. Fucoidan is derived from the cell wall matrix of brown algae and are rich in active substances, such as polysaccharides, terpenoids, proteins, polyphenols, sterols, multi-ring sulfur compounds, macrolides, and trace elements [4]. To extricate fucoidan from seaweeds, dilute acid, water, or alkali is generally used; however, these techniques require more time and larger quantities of reagents. As such, researchers have upgraded the conventional extraction techniques and standardized new techniques. The water molecules in cells are vibrated by microwave or ultrasound, causing the cells to split and thus enhance the coherence of conventional water extraction techniques. Enzyme-assisted extraction techniques have a high degree of coherence and precision and use enzymes to break down the cell wall [5].

The therapeutic benefits of fucoidan have attracted the interest of many researchers over the last 5–10 years. In many countries, such as Japan, China, and South Korea, brown seaweed is established as a local cuisine. Fucoidan is a complex sulfated heteropolysaccharide [6] comprising l-fucose-4-sulfate monosaccharides that consist of l-fucose and sulfate groups. Other monosaccharides such as uronic acid, galactose, xylose, mannose, rhamnose, glucose, arabinose, and xylose are also present. The two chain-forming structures of fucoidan are (1→3)-α-l-fucopyranose and α-l-fucopyranose linked by (1→3) and (1→4) [7]. Single and double substitutions in fucoidan occur at the C-2 and C-3 positions [8]. The factors on which the structure and composition of fucoidan depend are the species of seaweed, geographic site of collection, time of harvest, anatomical regions, and extraction procedures. Fucoidans are mined from other natural resources through the use of microwaves, hot water baths, or acid baths [9]. Extraction methods determine the bioactivity and molecular weight (Mw) of fucoidan, which may vary from 10,000–100,000 Da. Fucoidan is nontoxic, nonirritating, bioactive, and has many therapeutic applications [10]; thus, it has become a popular research topic in terms of its separation, purification, production, structural analysis, bioactivity, and oral absorption. This review highlights the bioactivity and the recently discovered cellular functions of fucoidan. Fucoidan is also important for the regulation of glucose and cholesterol metabolism [11]. Several studies have shown its antiviral, immunoregulatory, antitumor, anticoagulant, antithrombotic, anti-inflammatory, and antioxidant effects [12,13]. Important biological activities of fucoidan have been discovered by investigating these pathways. Furthermore, sulfate groups, Mw, natural sources, and extraction methods are some of the factors affecting the bioactivity of fucoidan [7]. In this review, we attempt to elucidate the mechanisms through which fucoidan hampers tumor growth and cancer cell activity and interacts with chemotherapeutic drugs and also discuss the hurdles faced in the anticancer application of fucoidan.

### 1.1. Fucoidan

Fucoidan was first sourced from species of brown algae, namely *Laminaria digitata*, *Ascophyllum nodosum,* and *Fucus vesiculosus,* in 1913. It is an extremely hygroscopic and negatively charged polysaccharide. The leaves of *L. digitata*, *A. nodosum*, *Macrocystis pyrifera,* and *F. vesiculosus* contain high levels of fucoidan [14]. Fucoidan is soluble in both water and acids, and recent studies have shown that it is beneficial in protecting against liver damage and urinary system failure [15,16].

### 1.2. Sources of Fucoidan

Sea cucumbers and brown algae are marine sources that harbor the sulfated polysaccharide fucoidan. Chorda filum, Hizikia fusiforme, Ascophyllum nodosum, Fucus evanescens, Fucus serratus, Fucus distichus, Fucus vesiculosus, Sargassum stenophyllum, Caulerpa racemosa, Kjellmaniella crassifolia, Dictyota menstrualis, Analipus japonicus, Padina gymnospora, and Laminaria hyperborea are some of the algae and invertebrates containing fucoidan, among which the content, type, and preferred method of extraction vary [17].

### 1.3. Structure of Fucoidan

**Figure 1:**
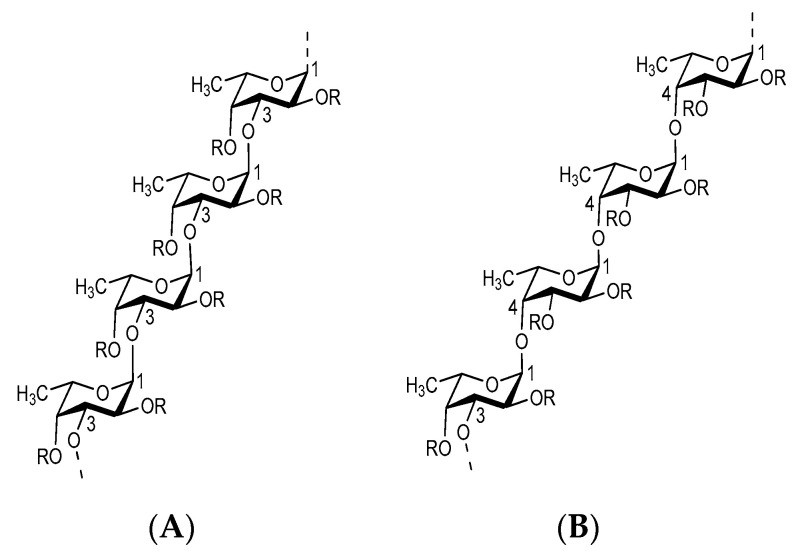
The chemical structures of fucoidan of two different backbones (**A**,**B**). R shows the potential places for attachment of carbohydrate (α-l-fucopyranose and α-d-glucuronic acid) and noncarbohydrate (sulfate and acetyl groups) substituents, adapted from [21].

Fucoidan, a fucose-enriched sulfated polysaccharide, is mainly extracted from the extracellular matrix of brown algae. In different species of brown algae, fucoidan consists of L-fucose and sulfate groups and one or more small xylose, galactose, mannose, rhamnose, glucuronic acid, glucose, arabinose, and acetyl groups [18]. Galactofucan fucoidan is a monosaccharide composed of galactose and fucose, similar to rhamnofucan (rhamnose and fucose) and rhamnogalactofucan (rhamnose, galactose, and fucose). Variation among the different seaweeds can be observed by examining the structure of fucoidan. However, fucoidan usually has two types of homofucoses; type one (I) comprises repeated units of (1→3)-l-fucopyranose, and the second type (II) encompasses alternating and recurring units of (1→3)- and (1→4)-l-fucopyranose [19,20]. The chemical structure of fucoidan is shown in Figure 1. 

### 1.4. Dosage and Course of Administration

The dosage of fucoidan varies greatly between different studies because of the different sources and decontamination techniques that are used [22]. Alwarsamy et al. found that fucoidan arrests 50% of cell reproduction in A549 lung cancer cells after 48 h of treatment with 100 µg/mL fucoidan [23]. The antitumor activity of fucoidan was studied in C57 *BL*/6 mice with Lewis lung adenocarcinoma. The results showed that there was no considerable impact on tumors when mice were injected with 25 mg/kg fucoidan. Meanwhile, mice could endure a repeated dosage of 10 mg/kg of fucoidan, and the drug revealed remarkable antitumor (inhibited tumor growth by 33%) and antimetastatic activities (29% reduction) [24]. Intraperitoneal injection and/or administration of fucoidan through food, gavages, subcutaneous injection, and intravenous injection have also been thoroughly researched [25,26,27,28,29].

## 2. Anticancer Potential of Fucoidan: Insights from Recent Studies

Cancer is a composite disease with unprecedented cell growth. Factors such as sepsis, smoking, occupational exposure, environmental pollution, obstructive diet, and hereditary components influence the complex procedure of the growth and development of the human body [30]. Tumor cell propagation and maintenance are generally associated with uncommon subcellular signal transduction and the uninterrupted sustenance of cellular growth [31]. For example, because of its participation in numerous cellular functions involving mRNA, cell cycle regulation, gene copy, apoptosis, autophagy, and metabolism, the P13K-AKT-mTOR signaling pathway is often engaged. Surgery, radiotherapy, and chemotherapy are the main dependable lines of cancer treatment [32,33,34]. However, the side effects of these treatments are significant, and the therapeutic outcomes are limited. It has been observed that a few of the inherent intrinsic signaling pathways can hinder or slow carcinogenesis at different phases and they exhibit characteristics such as explicit targeting, reduced cytotoxicity, and the induction of cancer cell apoptosis [35,36,37,38].

Fucoidan has been used as a medicinal food supplement in Asia owing to its medicinal function and anticancer ability [39,40], which has been extensively studied since the 1980s [25,41]. Several studies have shown that fucoidan can act against cancer through cell cycle arrest, thereby hindering angiogenesis by inducing apoptosis or activating natural killer (NK) cells or macrophages [42,43]. Additionally, fucoidan has countless superior biological activities, which include anti-inflammatory, antioxidant, anticlotting, antithrombotic, antiviral, anti-angiogenesis, and anti-*Helicobacter pylori* activities [6,21,44,45,46,47]. Natural extracts are associated with high biological activity, a wide range of sources, low drug resistance, and a low number of side effects compared with chemically synthesized drugs; therefore, natural extracts are actively being researched as novel antitumor drugs or as complementary drugs in combination with conventional antitumor drugs [48]. Fucoidan has a strong natural antioxidant activity and can substantially scavenge surplus free radicals. In one study, the low-molecular-weight fucoidan (LMWF) was processed and the fractions DF1, DF2, and DF3 were obtained [49], all of which showed superoxide anion radical scavenging activity. It has been seen that the antiviral action of fucoidan is strongly related to its sulfate content [50]. Its antiviral activity increases with an increase in the mass fraction of the constituting sulfate groups [51]. Nonetheless, the molecular weight and structure of the fucoidan acquired via various extraction techniques are distinct, and these factors have unquestionable effects on fucoidan’s biological activities [6,17].

### 2.1. Fucoidan Modulates Apoptosis and Cell Cycle

Necessary processes, such as embryonic development and homeostasis in organisms, are maintained by apoptosis, also known as programmed cell death. This section highlights how malignant or cancer cells undergo apoptosis in multiple ways after stimulation with fucoidan; these multiple pathways include the caspase system, cell cycle checkpoints, and internal and external pathways [52]. At a concentration of 1.0 mg/mL, fucoidan derived from *C. okamuranus* increased the G0/G1-phase population fraction of Huh7 hepatocarcinoma cells. This mechanism was followed by a decrease in the S-phase fraction, suggesting that fucoidan may cause cell cycle arrest in the G0/G1 phase [53]. Zhang et al. demonstrated that when high-molecular-weight fucoidan was extracted from *Cladosiphon novae-caledoniae kylin* and then digested with glysidases, it produced LMWF. LMWF consists of a low-molecular-weight digested fraction (72%) and an undigested fraction of less than 28%. LMWF consists mainly of fucose, xylose, and mannose. Furthermore, LMWF complexed with tamoxifen, cisplatin, or paclitaxel shows cell growth inhibition, cellular apoptosis, and arrest of the cell cycle in the human breast cancer cell line MCF-7/ MDA-MB-231. The study revealed that in breast cancer cells, phosphorylation of different proteins, elevation the reactive oxygen species (ROS) levels, and reduced glutathione (GSH) levels were all crucial in cancer cell apoptosis [54].

Researchers conducted comparative apoptosis studies and found that type II fucoidan isolated from *F. vesiculosus* showed similar apoptosis induction activities through caspase-8 and -9 activation in MCF-7 and HeLa cells to the low-molecular-weight type I fucoidan derivatives [55,56,57,58]. Fucoidan is a potential adjuvant for treating melanoma. Although therapeutic strategies involving combined therapies exist, their efficacy depends on several factors, including the overall health of the patient, the stage of metastases, and the melanoma location [59]. However, the effectiveness of these treatments may be reduced slightly because of the progression of new resistance mechanisms. Therefore, new therapeutic targets for melanoma are urgently needed. For example, *F. vesiculosus* fucoidan showed inhibitory effects on cell proliferation and apoptosis induction in B16 melanoma cells [60]. Fucoidan inhibits tumor cells by activating apoptosis and is therefore a potential therapeutic agent. Several studies have been conducted to develop fucoidan as an anticancer therapeutic by combining it with other anticancer agents [61,62,63]. However, more cancer studies are needed, particularly considering the discrepancies in the results of animal studies and human clinical trials, which can be caused due to the way the human body absorbs and processes fucoidan [52,64,65,66]. The next section briefly describes the known anticancer mechanisms of fucoidan.

### 2.2. Possible Pathways Involved in the Anticancer Action of Fucoidan

The anticancer mechanism of fucoidan has been shown to primarily include four elements, according to previous reports:

- Inhibition of normal mitosis and cell cycle regulation:

Fucoidan reduces cancer cell proliferation by inhibiting normal mitosis and cell cycle regulation [67]. When fucoidan was injected into C57 mice with transplanted Lewis lung adenocarcinoma cells, it was observed that the number of tumor masses and lung metastases was significantly lower than that in cyclophosphamide treated mice, indicating that metastasis and tumor cell growth are effectively inhibited by fucoidan [24].

- Activation of tumor cell apoptosis signals:

Fucoidan leads to the activation of tumor cell apoptosis signals, leading to anticancer effects [68]. HT-29 and HCT116 human colon cancer cells were cocultured with fucoidan extracted from *Fucus vesiculosus*. The results showed that fucoidan induced caspase-3, -7, -8, and -9 activation, chromatin condensation, and poly (ADP-ribose) polymerase (PARP) cleavage [69].

- Inhibition of vascular endothelial growth factor (VEGF) formation:

VEGF formation can be inhibited by fucoidan, which leads to angiogenesis suppression, interruption in the supply of nutrients and oxygen to the tumor, tumor volume reduction, and inhibition of the spread of cancer cells [70,71]. Fucoidan was administered to mice implanted with Lewis lung cancer cells, and the results showed reduced VEGF levels in serum and lung tissue compared to those in non-FUCs [7]. Fucoidan or fucoidan persulfate can inhibit VEGF165 mitosis and chemotaxis in human umbilical vein endothelial cells by inhibiting VEGF165 at cell surface receptors [71,72]. Fucoidan also inhibits cell-induced neovascularization of human prostate cancer (DU-145) as observed in mice with transplanted B16 melanoma cells. Thus, these results showed that the antitumor activity of fucoidan is related to its anti-angiogenic effect [73,74].

- Stimulation of NK cells and T lymphocytes:

Fucoidan activates the immune system by elevating the actions of NK cells and T lymphocytes to target cancer cells. Mice transplanted with NB4 (acute promyelocytic leukemia cells) were fed fucoidan, which led to an increased killing activity of cancer cells by NK cells [75]. Table 1 summarizes the in-vitro effect of fucoidan isolated from various sources of marine algae on cancer cells.

### 2.3. Effectiveness of Fucoidan against Colon Cancer

Colon cancer is the most common form of cancer worldwide. When the human colon cancer DLD-1 model was administered with fucoidan extracted from brown alga *Saccharina cichorioides,* tumor cell proliferation was inhibited by the inhibition of the epidermal growth factor activity [76,91]. HT-29 and HCT116 cell lines undergo fucoidan-induced apoptosis, which is regulated by mitochondrial-mediated and receptor-mediated apoptotic pathways [69]. Thinh et al. reported three fucoidan fractions (SmF1, SmF2, and SmF3) extracted from *Sargassum mcclurei* where all fractions were found to be less cytotoxic and exhibited inhibition of colony formation in colon cancer DLD-1 cells [92]. HT-29 cell death is induced by administration of fucoidan, the probable reason for which could be the downregulation of IGF-IR signaling via the IRS-1/PI3K/AKT pathway [93].

Mice with colon tumors were administered low-, medium-, and high-molecular-weight fucoidan. Medium-molecular-weight fucoidan was observed to significantly inhibit tumor growth. The results also showed that the survival time of mice in the fucoidan-treated group was significantly higher than that of the mice in the control group, and there was a rapid increase in the number of NK cells in the spleen of the mice [27].

### 2.4. Therapeutic Potential of Fucoidan against Breast Cancer

Previous studies reported that apoptosis was induced by fucoidan in MCF-7 cells in a caspase-8-dependent pathway, along with chromatin condensation and nuclear DNA fragmentation [78,94,95]. When treated with fucoidan, T-47D cell proliferation was effectively inhibited and fucoidan posed very low toxicity to mouse epidermal cells [80]. MCF-7 cells treated with fucoidan from *Undaria pinnatifida* in New Zealand have been found to significantly suppress tumor cell proliferation with low cytotoxicity against normal tissue cells. The 3-[4,5 dimethylthiazol-2-yl]-2,5-di-diphenyltetrazolium bromide (MTT) method has been used by researchers to confirm the reduction in the number of viable cells by fucoidan [63]. The overall results of the respective study showed that fucoidan arrests the G1 phase by regulating apoptosis-related gene expression and the cell cycle. Fucoidan can effectively reverse the epithelial–mesenchymal transition (EMT) induced by TGFβ receptors (TGFR). This may lead to the upregulation and downregulation of epithelial and interstitial markers, respectively [96]. Fucoidan may inhibit the growth of MDA-MB-231 cells by reducing the expression of the transcriptional suppressors *Snail*, *Slug*, and *Twist* [96]. An in vivo study involving the administration of fucoidan to mice with 4T1 showed that the tumor volume was significantly reduced in the fucoidan-treated group compared with that of the control group injected with phosphate-buffered saline. This study showed that fucoidan effectively inhibited 4T1 cell proliferation and metastasis [97]. Fucoidan, when combined with cisplatin, doxorubicin, and taxol, increased the cytotoxicity against MCF-7 breast cancer cells and thus it could be a promising compound in combination therapy [98]. *Cladosiphon okamuranus* extracted fucoidan in combination with coral-like Pt nanoparticles has proved to be a potential therapeutic agent against multidrug resistant breast cancer by multiple pathways such as anti-angiogenesis, interfering with metastasis, immune activation, and cell apoptosis [99].

### 2.5. Protective Effect of Fucoidan on Hepatoma Cells

Duan et al. showed that fucoidan plays a significant role in inhibiting cell proliferation in BEL-7402 and LM3 cell lines through the p38MAPK/ERK pathway [82]. Fucoidan administration results in the upregulation of NDRG-1/CAP43, mediated by the inhibition of hepatocarcinoma cells. Fucoidan may also lead to a reduction in hepatoma cell metastasis through the upregulation of p42/44 MAPK-mediated vacuolar membrane protein 1 (1VMP-1), inhibition of caspases-7 and 8, and activation of the Fas-related death domain. Fucoidan showed antimetastatic activity in a MH134 cell model of liver metastasis [100].

Human hepatoma cells (SMMC-7721) exhibited significant growth inhibition and apoptosis induction following treatment with fucoidan. GSH consumption is associated with fucoidan-induced SMMC-7721 cell apoptosis. Treatment with fucoidan leads to increased levels of ROS in cells, along with mitochondrial damage and mitochondrial membrane potential (MMP) depolarization. Thus, the evidence clearly shows that fucoidan may induce apoptosis in human hepatocellular carcinoma SMMC-7721 cells via a ROS-mediated mitochondrial pathway [83].

Few researchers have reported the upregulation of microRNA-29b (miR-29b) in human HCC and the inhibition of its downstream target DNA methyltransferase 3B (DNMT3B) by the administration of a fixed dose of fucoidan [101]. Inhibition of DNMT3B leads to suppression of mRNA and tumor metastasis suppressor gene 1 (MTSS1). In hepatoma cells, fucoidan administration may downregulate the transforming growth factor (TGF) receptor and SMAD signaling, which leads to the inhibition of extracellular matrix degradation and a reduction in the invasive activity of HCC cells [101,102].

### 2.6. Fucoidan Exhibits Antileukemia Effects

Various studies on the antileukemic effect of fucoidan have yielded good results, and researchers have investigated the signaling pathway for fucoidan-mediated apoptosis [103,104]. HL-60 cells treated with fucoidan showed the activation of caspases-3, -8, and -9 and a change in the permeability of the mitochondrial membrane [85]. Mitogen-activated protein kinase p38 (MAPK) and the MAPK inhibitor p38 may be effectively activated by fucoidan [104]. Activation of p38 MAPK may play an important role in fucoidan-induced apoptosis. Moreover, the same study showed that increased apoptosis is associated with caspase hydrolases, Bid cleavage, Bax insertion into mitochondria, cytochrome c release from mitochondria, and membrane potential disruption in U937 cells [104].

**Figure 2:**
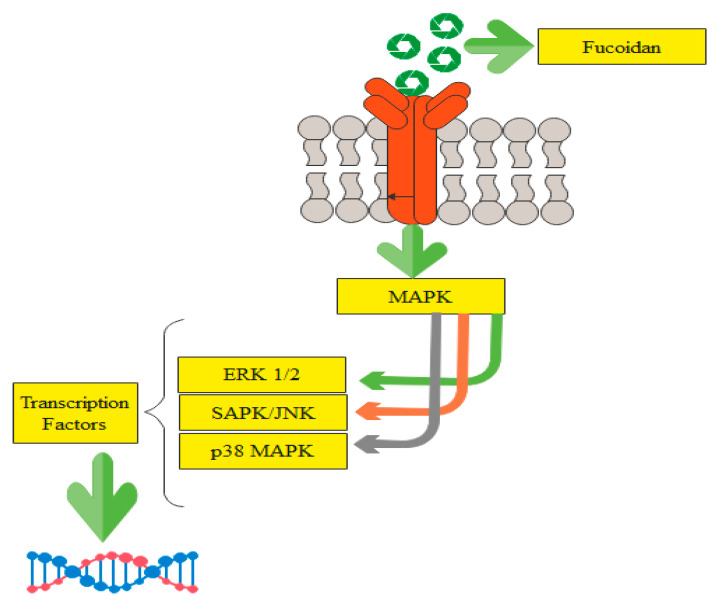
Fucoidan binds to specific types of receptors in macrophage cell membranes and activates MAPKs, which further prompt the activation of transcription factors, adapted from [25].

Figure 2 depicts the fucoidan pathway in macrophage cells, which causes the generation of signaling proteins and further stimulates immune cells to destroy cancer cells.

Researchers have investigated the cytotoxicity and antitumor activity of fucoidan in human acute myeloid leukemia cells [85,105]. The results of these analyses revealed that fucoidan significantly inhibits the proliferation and apoptosis of NB4 and HL60 cells by both endogenous and exogenous pathways [85].

In a study on NB4-transplanted mice, researchers observed that fucoidan can delay xenograft tumor growth and increase the cytolytic function of NK cells [85]. Researchers have also studied the anticancer activity of fucoidan in large B-cell lymphoma cells (DL-BCL); [106] the results showed a loss of MMP in the lymphoma cells along with cytochrome c release from their mitochondria and induction of lymphoma cell-specific apoptosis.

### 2.7. Therapeutic Effects of Fucoidan against Human Bladder Cancer

Reports have confirmed that some fucoidan-induced apoptosis effects have been observed after treatment with fucoidan, including mitochondrial dysfunction; increased Bax/Bcl-2 expression; Bid cleavage; Fas upregulation; sequential activation of caspases-8, 9, and 3; and decreased PARP degradation and IAP expression. Park et al. treated human bladder cancer cells with fucoidan and found that fucoidan inhibited tumor growth by enhancing the expression of cyclin-dependent kinase 1 inhibitors [107]. One another study reported the effect of fucoidan on bladder cancer cell growth by inducing G1 cell cycle arrest, which resulted in a reduced viability of EJ human bladder cancer cells [108]. The same group revealed that the fucoidan-induced arrest was associated with increased CDK inhibitor expression and the dephosphorylation of pRB. Other effects of fucoidan have also been reported, such as MMP loss and the release of cytochrome c from mitochondria to induce apoptosis [95,104]. These observations have shown that fucoidan plays an important role in the interaction between endogenous and exogenous caspase-dependent apoptotic pathways. Human telomerase reverse transcriptase enzyme, primary tumor transcription factor, and promotional protein 1 expression were reportedly reduced in fucoidan-treated human bladder cancer cells [109]. They also found that by inhibiting PI3K/Akt signaling pathway activation, fucoidan increases apoptosis and decreases telomerase activity, which is mediated by ROS-dependent PI3K/Akt pathway inactivation [109].

### 2.8. Action of Fucoidan against Lung Cancer

A previous study demonstrated that the administration of fucoidan led to an elevation of viral symptoms in C57BL/6 mice, which resulted in the inhibition of lung metastases in mice with transplanted Lewis lung cancer cells [110]. Previous studies have used C57BL/6 mice inoculated with Lewis lung cancer cells to investigate the combined effect of cyclophosphamide and fucoidan used as an adjuvant. The results obtained from the above experiment showed that repeated administration of fucoidan showed only an antimetastatic effect, and not an antitumor effect, with cyclophosphamide [24,111].

Moreau et al. found that when the human carcinoma line NSCLC-N6 was treated with fucoidan extracted from *Bifurcaria bifurcate,* cancer cells were irretrievably obstructed [88]. Fucoidan-treated A549 human lung cancer cells showed significant inhibition of tumor cell proliferation with low cytotoxicity against normal tissue [56]. Fucoidan from *Undaria pinnatifida* has been used for treating A549 cells, has antiproliferative activity, and regulates MAPK p38 [87]. Fucoidan extracted from *Turbinaria conoides* leads to a dose-dependent reduction in the survival rate of A549 cells, but it is not cytotoxic to a noncancerous human skin tissue keratinocyte (HaCaT) cell line [23].

Fucoidan combination therapy with anticancer drugs has been thoroughly reviewed by several researchers, and these reports have been found to be beneficial in terms of cancer prevention. Researchers have investigated the effect of fucoidan on sequential treatment (based on cisplatin), demonstrating that fucoidan upregulates the expression of cleaved caspase-3 and PARP [90]. A study in C57BL/6 mice transplanted with LLC-1 cells revealed that the combination of cisplatin and fucoidan was more effective in suppressing tumor volume than was the individual administration of each drug. In mice, fucoidan has been shown to suppress new blood vessels induced by sarcoma 180 cells [71]. The study demonstrated that fucoidan exhibited an effective antitumor effect owing to its anti-angiogenic capacity. Qiu et al. investigated a combination study of fucoidan and gefitinib in tyrosine kinase inhibitor-resistant lung cancer cell lines by alleviating TGF-mediated slug expression, which was found to be a potent therapeutic approach. Previously, the combination of gefitinib and fucoidan significantly inhibited lung cancer cell viability by inducing an apoptotic response [112]. In another study, the combination treatment with GIV-A (fucoidan) and 5-FU significantly repressed the lung metastases. Furthermore, GIV-A improved the grade of spleen cell-mediated red blood cell hemolysis in sheep, indexes of the spleen and thymus, the number of spleen cells, and reinstated the 5-FU’s suppressive effect. The study documented an alteration in the levels of Thy1.2-, L3T4- and asialo GM1-positive cells; activation of C3 and macrophages; and a lowering of the liver drug-metabolizing system [113].

### 2.9. Fucoidan and Miscellaneous Cancer Therapies

Oral administration of fucoidan (5 mg/kg) effectively inhibited tumor growth in mice grown with B16 melanoma cells. Oversulfated fucoidan has been found to suppress VEGF expression, inhibit neoplastic angiogenesis, and appears to be more effective than fucoidan [71]. Fucoidan-mediated treatment of the melanoma RPMI-7951 cell line has shown that fucoidan can regulate tumor cell turnover and affect tumor cell division [76]. PC-3, a human prostate cancer cell line, was cultured with fucoidan extracted from *Undaria pinnatifida* in a dose-dependent manner (μg/mL) [114]. Fucoidan administration led to the ERK1/2 MAPK-mediated inhibition of the p38 MAPK and PI3K/Akt signaling pathways, promoted PC-3 apoptosis, and protected against uncontrolled cancer division.

DU-145 cells were treated with fucoidan at a dose of 100–1000 μg/mL. Fucoidan inhibited the proliferation and functioning of DU-145 cells and the migration and processing of the cells in the matrix [73]. In an in vivo experiment, DU-145 cells were injected into mice to create cancer xenograft models [115].

Oral gavage at a dose of 20 mg/kg fucoidan for 28 days significantly suppressed tumor growth and angiogenesis, reduced hemoglobin content in tumor tissue, and decreased CD31 and CD105 mRNA expression [73]. In addition, activation of JAK, STAT3, VEGF, Bcl-kL, and cyclin D1 was significantly reduced after fucoidan treatment. Moreover, the results indicated that both the anticancer and anti-angiogenic effects of fucoidan could be mediated by JAK/STAT3 [73]. Hence, different doses and delivery routes may affect fucoidan metabolism in in vivo models and impact the possible treatment outcomes. Researchers have explored the potential mechanism of the antiproliferative effect of fucoidan on human gastric adenocarcinoma AGS cells [116]. The results showed that fucoidan can inhibit Bcl-2 and Bcl-xL expression and reduce MMP and the levels of protein polymerase (ADP-ribose). These data indicate that fucoidan can effectively inhibit AGS cell growth by stimulating autophagy and apoptosis. Bobiński et al. investigated activity of fucoidan on uterine sarcoma cell lines ESS-1 and MES-SA and the cancer cell lines SK-UT-1 and SK-UT-1B, and their toxic effects on human skin fibroblasts. The results showed that the viability of SK-UT-1, SK-UT-1B, and ESS1 cell lines was reduced following treatment with fucoidan, with no adverse effects on the proliferation of the adjacent non-cancer cells [117]. Thus, it was concluded that fucoidan not only affects cancer cell proliferation, but also potentially causes cytotoxic uterine cancer cell apoptosis.

There are various studies that explore the combined therapy of fucoidan with chemotherapy against different cancers. The fucoidan effects in combination with these drugs represent a novel approach for improving the side effects and ameliorating the immune response. The combined use of gemcitabine and cisplatin with low-molecular-weight fucoidan has been studied and showed an improvement of muscle atrophy in bladder cancer by inhibiting NF-kB mediated inflammation, activin A, and myostatin [118]. An another group investigated *Undaria pinnatifida* (UPF) or *Fucus vesiculosus* (FVF) in combination with paclitaxel and showed a possible antagonistic effect in breast cancer models (MCF-7 and ZR-75D) [119].

Fucoidan extracted from *Cladosiphon okamuranus* was administered in a combination treatment of chemotherapeutic drugs, namely oxaliplatin plus 5-fluorouracil/leucovorin (FOLFOX) or irinotecan plus 5-fluorouracil/leucovorin (FOLFIRI), and showed potential anticancer effects against recurrent colorectal cancer by lowering the cytotoxic effect of the chemotherapy drugs in patients, such as nausea, vomiting, stomatitis, diarrhea, liver dysfunction, etc. [120]. Further clinical trials and the development of fucoidan applications are required to address the safety concerns.

## 3. Reports on Human Consumption of Fucoidan

In recent years, there have been only a few studies on the possible systemic effects of oral fucoidan, and existing studies have mostly been conducted in mice. Few clinical studies on fucoidan have been reported because of the difficulty in ensuring the accuracy and representativeness of the study and quantifying the concentration of fucoidan in the body [17]. Fucoidan has not yet been approved as a medicinal product; therefore, large clinical trials cannot be performed.

By investigating many anticancer properties and mechanisms associated with fucoidan, researchers have found that its low toxicity and anti-inflammatory effects make it an ideal adjuvant therapeutic in the treatment of cancer. Hidenori et al. provided evidence that fucoidan acted as a potential anti-inflammatory agent in several patients with advanced cancer. Twenty patients with advanced cancer were selected for the study, in which oral fucoidan (4 g daily) was administered for at least four weeks. After two consecutive weeks of ingestion, there was a significant reduction in the levels of key proinflammatory cytokines, including interleukin 1-(IL-1β), IL-6, and tumor necrosis factor alpha (TNF-α), but no significant change was observed in patients’ quality of life, including the experience of fatigue [121].

In a 12-week randomized, double-blind, controlled study in patients with osteoarthritis, treatment efficacy was measured by evaluating osteoarthritis severity, liver function, cholesterol levels, hematopoietic function, and renal function to determine the safety of fucoidan administration and to carefully monitor its side effects. The results showed that 300 mg fucoidan is safe and well tolerated by humans [122]. However, the study reported that fucoidan did not significantly reduce the symptoms of osteoarthritis. In another study, researchers recruited 13 patients with HTLV-1-associated myelopathy/tropical spastic paralysis. The patients were administered 6 g fucoidan daily for 6 months. The associated results showed that previral DNA load in patients receiving fucoidan was significantly reduced by approximately 42.4% compared to that in the control group [123].

LMWF is a nutritional supplement that has been tested in patients with metastatic colorectal cancer as an adjunct to chemotherapy drugs and targeted drugs. The study was a double-blind, controlled trial involving approximately 54 patients. Twenty-eight subjects in the experimental group received 4 g fucoidan per day, and twenty-six subjects in the control group received 4 g cellulose per day. There was a significant difference in disease control rates between the experimental and control groups. This was the first clinical study to evaluate the effectiveness of LMWF as a supplemental therapy in patients with metastatic colorectal cancer [124].

A study was conducted in breast cancer patients to observe the effect of fucoidan derived from *Undaria pinnatifida* on the pharmacokinetics of hormone therapies such as those involving letrozole and tamoxifen. When patients were administered 1 g fucoidan daily for 3 weeks, the results revealed stable plasma concentrations of letrozole, tamoxifen, and the metabolites of tamoxifen after binding to fucoidan. Nevertheless, no significant difference in toxicity was observed during this period. These findings suggest that fucoidan can be used safely in conjunction with letrozole and tamoxifen [125].

Previous studies have asserted the pharmacokinetic properties of fucoidan by confirming its absorption using enzyme-linked immunosorbent assays (with specific antibodies) [126,127]. An observational study on healthy subjects who were administered or who consumed fucoidan showed that some amount of fucoidan was assimilated through endocytosis and could be detected in the blood and urine of the recruited subjects. Moreover, LMWF extracted from *S. japonica* resulted in both a higher absorption rate and bioavailability compared with those of the medium-molecular-weight fucoidan [128]. However, the biosafety and biodistribution of fucoidan still need to be explored further in humans.

## 4. Possible Side Effects of Consuming Fucoidan

Currently, there are very few studies on the side effects of fucoidan. Researchers have tested the toxicity of oral fucoidan in Sprague-Dawley rats. No major abnormalities in rat biomarkers were observed in mice administered 150–1350 mg/kg fucoidan daily for 28 days, and only female rats showed an increase in blood urea nitrogen [129]. Due to the adjuvant properties of fucoidan, it is used in combination therapy with anticancer drugs. Oh et al. reported that fucoidan alleviated the efficiency of lapitinib and exhibited antagonistic effects on the proliferation of some cancer cell lines [130]. In Ames tests, a concentration of 500 μL fucoidan per plate showed no significant effect on the induction of colony growth. However, after taking 2000 mg/kg fucoidan daily, the rats exhibited an elevation of thyroid weight. Fat metabolism and alanine transaminase levels have been reported to significantly change in rats [131]. Li et al. revealed that daily administration of 300 mg/kg/body weight fucoidan extracted from *Laminaria japonica* to rats for over 180 days did not prompt any detrimental side effects; nonetheless, a higher dose of 900–2500 mg/mL caused coagulopathy and noticeably reduced blood clotting time [132]. A human study reported that 4 of 17 patients taking approximately 6 g fucoidan per day had diarrhea, and their condition was stable after discontinuing fucoidan intake. These findings suggest that fucoidan may be harmful to the liver. However, no significant research has been carried out, and it remains impossible to adequately assess the adverse effects of fucoidan [133]. Moreover, one case study suggested that one woman with excessive dietary intake of seaweeds or “Nori” had carotenodermia and an orange-yellow skin color [134].

## 5. Conclusions

Several studies have demonstrated the anticancer effect of fucoidan, including inhibition of the growth of various cancer cells, metastasis, angiogenesis, and induction of apoptosis in vitro and in vivo. Additionally, when administered with chemotherapy and radiotherapy drugs, fucoidan acts as an immunomodulatory molecule and reduces side effects, thus showing great potential in cancer treatment. However, because of the lack of information on drug interactions between fucoidan and conventional anticancer drugs, there is little clinical data on fucoidan.

More experimental studies are needed to explore the mechanisms involved in cancer treatment. Fucoidan may become an appropriate and natural therapeutic or adjunctive antitumor drug, providing new directions for the development of new anticancer drugs in the future.

## Figures and Tables

**Table 1:** Effect of fucoidan on cancer cells in-vitro.

Effect of Fucoidan	Cell Type	Fucoidan Source	Study Findings	Mechanism of Action	Ref.
**Colon cancer cells**	DLD-1	*Saccharina cichorioides*	EGF receptor binding inhibition with EGF and colony formation inhibition	Inhibits cell proliferation	[76]
	HT-29 HCT-116	*Fucus vesiculosus*	Downregulating the PI3K-Akt-mTOR pathway, Activation of Caspase-8, 9, 7, 3 activation, ↑ PARP, Bak, Bid, Fas, ↓ Mcl-1, survivin, XIAP	Induces cell apoptosis	[69,77]
	WiDr LoVo	*Undaria pinnatifida*	Less cytotoxic and can be used as functional food in cancer treatment	Suppresses cell proliferation	[56]
**Breast cancer cells**	MCF-7	*Fucus vesiculosus*/ *Cladosiphon okamuranus*	PARP cleavage Caspase-7,8,9 ↑ Cytochrome C, Bax, Bid ↑ Modulating E-cadherin and MMP-9 expression inhibition of tumor cell migration	Induces tumor cell apoptosis and inhibit proliferation	[78,79]
	T-47D	*Saccharina japonica*	Cytotoxicity against human breast cancer	Inhibits cell proliferation and colony formation	[80]
	MDA-MB-231	*Fucus vesiculosus*	Activation of caspases and mitochondrial dysfunction along with altering Ca(2+) homeostasis, cytochrome c release	Cancer cell death	[81]
**Hepatoma carcinoma cells**	BEL-7402 LM3	*Fucus vesiculosus*	Pathways targeted were p38 MAPK/ERK pathways, PI3K/Akt, and upstream kinases. Alteration in phosphorylation of p38 MAPK and ERK	Promotes apoptosis, inhibits cell proliferation	[82]
	SMMC-7721	*Undaria pinnatifida*	Livin, XIAP mRNA ↓ Caspase-3, -8, -9 ↑ Bax-to-Bcl-2 ratio ↑ Cytochrome C ↑ Quantity of mitochondria ↓ ROS ↑ Depolarization of the MMP	Induces cell apoptosis	[83]
	Huh-7 SNU-761 SNU-3085	*Fucus vesiculosus*	A molecule called ID-1, which was significantly suppressed, Down-regulation of ID-1 s was dependent on NDRG-1/CAP43	Anti-metastatic effect	[84]
**Leukemia cells**	NB4 HL60	*Fucus vesiculosus*	Caspase-3, 8, 9 ↑ PARP cleavage Bax ↑ Activation of ERK1/2, AKT ↓ NK cell ↑	Inhibits cell proliferation, induces cell apoptosis	[85]
	U937	*Cladosiphon okamuranus*	Apoptosis via caspase-3 and -7 activation-dependent pathway PARP cleavage	Inhibits cell proliferation, induces cell apoptosis	[86]
**Lung cancer cells**	A549	*Undaria pinnatifida*	Bcl-2, p38, Phospho-PI3K/Akt, procaspase-3 ↓ Bax, caspase-9, Phospho-ERK1/2-MAPK ↑ PARP cleavage	Inhibits cell proliferation, induces cell apoptosis	[87]
	NSCLC-N6	*Bifurcaria bifurcata*	Irreversible growth arrest	Inhibits cell proliferation	[88]
	Lewis lung carcinoma cells	*Fucus vesiculosus*	PI3K-Akt-mTOR pathway ↓ Caspase-3 ↑ Inhibition of VEGF, MMPs	Inhibits metastasis and induce apoptosis of cancer cells	[89]
	H1975	*Fucus vesiculosus*	Caspase-3 ↑ PARP cleavage TLR-4 mediated endoplasmic reticulum stress	Increases inhibition rate, induces cell apoptosis	[90]

This table was modified and redrawn from Lin et al. 2020 [25].

## Data Availability

Not applicable.
